# ParticipACTION: Baseline assessment of the capacity available to the 'New ParticipACTION': A qualitative study of Canadian organizations

**DOI:** 10.1186/1479-5868-6-87

**Published:** 2009-12-09

**Authors:** Guy Faulkner, Cora McCloy, Ronald C Plotnikoff, Adrian Bauman, Larry R Brawley, Karen Chad, Lise Gauvin, John C Spence, Mark S Tremblay

**Affiliations:** 1 University of Toronto, Faculty of Physical Education and Health, 55 Harbord Street, Toronto, ON, M5S 2W6, Canada; 2 University of Alberta, Centre for Health Promotion Studies - School of Public Health, and Faculty of Physical Education and Recreation, University of Alberta. Edmonton, AB, TG6 2T4, Canada; 3 University of Sydney, School of Public Health, K25 - Medical Foundation Building, The University of Sydney, Sydney, NSW 2006, Australia; 4 University of Saskatchewan, College of Kinesiology, PAC 300 87 Campus Drive, Saskatoon, Saskatchewan, S7N 5B2, Canada; 5 Office of the VP - Research, University of Saskatchewan Box 5000 RPO University 110 Gymnasium Place, Saskatoon, SK S7N 4J8, Canada; 6 Université de Montréal, Faculty of Medicine, Department of Social & Preventive Medicine, GRIS (Groupe de recherche interdisciplinaire en santé), Centre de recherche Léa-Roback sur les inégalités sociales de santé de Montréal, Centre de recherche du Centre Hospitalier de l'Université de Montréal (CRCHUM), POBox 6128, Downtown Station, Montreal, P.Q. H3C 3J7, Canada; 7 University of Alberta, Faculty of Physical Education and Recreation, E 488 Van Vliet Centre, Edmonton, AB, T6G 2H9, Canada; 8 Healthy Active Living and Obesity Research Group, Children's Hospital of Eastern Ontario Research Institute, Ottawa, ON, K1H 8L1, Canada

## Abstract

**Background:**

Evaluation of the original ParticipACTION campaign effects focused on individual awareness, recall, and understanding. Less studied has been the impact such campaigns have had on the broader organizational capacity to mobilize and advocate for physical activity. With the relaunch of ParticipACTION, the purpose of this study was to qualitatively explore baseline organizational capacity to promote physical activity messages, programs, and services within the Canadian context.

**Methods:**

Using a purposeful sampling strategy, we conducted semi-structured telephone interviews with 49 key informants representing a range of national, provincial, and local organizations with a mandate to promote physical activity. Interview data were analysed using a thematic analytic approach.

**Results:**

Key informants painted a generally positive picture of current organizational capacity to promote physical activity messages, programs, and services in Canada. Will and leadership were clear strengths while infrastructure limitations remained the greatest concern. Some specific challenges included: 1) funding issues: the absence of core funding in a climate of shifting funding priorities; 2) the difficulty of working without a national physical activity policy (lack of leadership); 3) inconsistent provincial and educational sector level policies; and 4) a persistent focus on obesity rather than physical inactivity.

**Conclusion:**

The data generated here can be utilized to monitor the future impact of ParticipACTION on enhancing and utilizing this organizational capacity. A range of indicators are suggested that could be used to illustrate ParticipACTION's impact on the broad field of physical activity promotion in the future.

## Background

Most evaluative studies of social marketing campaigns and organizations promoting physical activity have tended to focus specifically on individual effects and message recall [1]. Bauman and colleagues [2] highlight the need for evaluation focusing on the impact of such campaigns on 'gatekeeper' groups such as policy makers, health professionals and advocates, and industry [3]. Less studied has been the impact these campaigns have on the broader organizational climate to mobilize and advocate for physical activity or on how existing organizational capacity is utilized by a new social marketing initiative to achieve its own goals.

We suggest that increases in physical activity promotion, advocacy, and delivery capacity (intra-organizational, extra-organizational and inter-organizational) may be a critical intermediate level outcome of the new ParticipACTION and its future initiatives. The process of building capacity is an important dimension that fundamentally adds to the actual outcomes of health promotion [4]. The achievement of changes in long term outcomes, such as increases in population levels of physical activity, often takes years. The tracking of intermediate level outcomes, that indicate long term progress is being made, is therefore essential. Indeed, a broad objective of the original ParticipACTION was to influence decision makers and organizations to develop friendly infrastructures and supportive environments for physical activity with the consensus being that the original campaign was successful in creating "a more receptive climate for the institutional changes needed to help Canadians adopt physical activity" ([5], page S24). However, no systematic evaluation was conducted to support this conclusion.

At the start of this current study, the new ParticipACTION was launched and prominently listed objectives explicitly related to capacity building such as becoming a leading, national catalyst; a communicator of practical information; motivator of coordinated partnership actions; and, contributing to community capacity building (see [6]). Identifying current capacity then becomes important in providing a basis for evaluating how ParticipACTION uses or influences opportunities or resources (e.g., organizational capacity) that may affect future physical activity levels of Canadians (e.g., changes in federal policy or school curriculum policy). Accordingly, two inter-related and sequential studies were conducted including 1) a web-based survey of national physical activity and health organizational capacity (see [7]) and then 2) the current one which is an interview-based qualitative study with purposefully selected key informants drawn from the web-based survey. Qualitative approaches are particularly appropriate for exploring the process of capacity development because change may occur in unanticipated ways [8].

### Organizational Capacity

**Figure 1:**
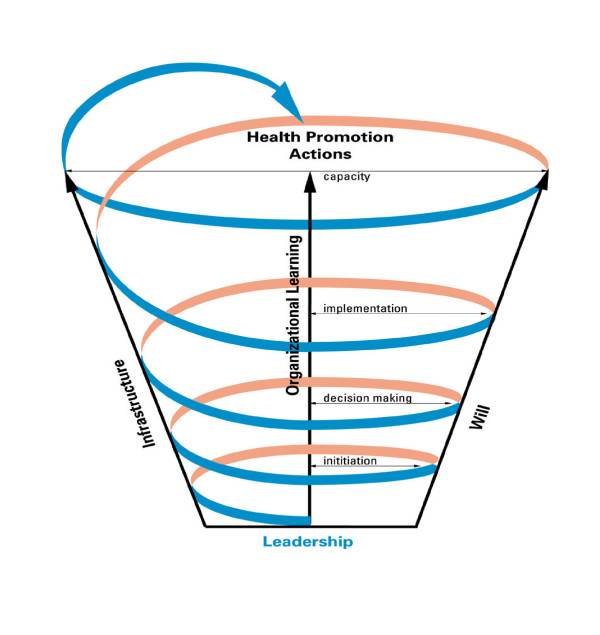
**Model of capacity building for health promotion (**[10]; **permission to reprint provided by SAGE)**.

Knowledge about organizational capacity and its development is limited [9] although evidence suggests that key components of organizational capacity include coalition building, networking, planning, management, delivery and evaluation of programs, and acquisition and availability of resources to achieve mandates. Smith and colleague's [10] (see Figure 1) theoretical model is an existing framework for examining organizational capacity and was chosen in this study as the theoretical lens for exploring perceptions of current capacity for promoting physical activity in Canada. The model includes three capacity indicators: leadership (e.g., the process of developing partnerships, collaborations, and linkages within the community); will/policy making (the process of developing vision, mission, and political will of the target community to implement and sustain a health initiative); and, infrastructure (e.g., the process of developing a supportive system and organization in the health sector, and the skills, knowledge, and resources for health promotion). Drawing on experiences from the Alberta Heart Health Project, it was found that overall capacity for health promotion was strengthened when these individual dimensions, or their interaction, were increased [11]. Further research informing the present study included the work of Joffres and colleagues [12] who offer a broader theoretical framework for organizational capacity that captures much of Smith et al's [10] capacity elements, referring to these as "internal context" but situates these in an "external context" (political climate, community awareness and interest, existing inter-organizational partnerships) which may challenge development within those dimensions.

### The current investigation

The purpose of this study was to explore baseline organizational capacity to promote physical activity within the Canadian context - by capturing a descriptive "snapshot" of organizational activities in the field of physical activity promotion in terms of will/policymaking, infrastructure, and leadership. This is the first qualitative study to our knowledge that establishes the foundations to examine subsequently the impact of a social marketing organization on physical activity promotion capacity at the organizational level.

## Methods

### Sampling

This qualitative, interview-based study involved 49 key informant interviews. Key informants were defined as individuals who have extensive involvement with, and knowledge of, their organization in relation to physical activity promotion. Informants were selected from a wide range of organizations engaged in physical activity promotion. Recruitment was linked to completing the web-based survey previously reported (for sampling details see [7]). At the end of the survey, participants (n = 268) had the option of providing consent for a follow-up interview. A subsample of 68 agreed to be contacted (response rate = 25.4%) and 54 were selected and sent email invitations: 34 agreed to an interview (cooperation rate = 63.0%). Since ensuring heterogeneity was a key consideration, participants were invited on the basis of ensuring that all province/territories, and organizational sectors and mandates were sampled. Some potential interview participants (n = 14) were not invited because there was over-representation from a) a particular province (Ontario), or b) the educational sector. To ensure broader representation, a further 15 participants who had not taken part in the initial quantitative survey, were invited to participate to ensure adequate coverage of all mandates and organizational characteristics [13]. However, there was still an under-representation of informants from Quebec, and private-for-profit organizations reflecting the initial survey responses (see [7]).

Ultimately, 49 individuals were interviewed and this sample size is comparable to qualitative components of larger projects examining capacity for heart health promotion [14]. The organization level breakdown included: 24 national organizations with some overlap between provincial and national levels in three of these groups (coded N1-N24); 19 provincial level organizations (coded P1-P19), and; 6 local level organizations (coded L1-L6).

### Data Collection

Semi-structured telephone interviews were conducted, ranging from 30 to 60 minutes. An interview guide was developed to explore perceptions of current capacity and was pilot tested with two individuals working in the physical activity field (for a national physical activity organization and a provincial educational organization respectively). Pilot testing generated constructive feedback on the content and the format of the interview. As others have reported, there can be difficulty in talking about "nebulous concepts such as collaborative building and advocacy" ([12], page 46) and both individuals found describing the capacity of their organization difficult without the addition of explicit prompts in suggesting examples related to will/policymaking, infrastructure, and leadership.

Accordingly, the interview guide (available on request from the first author) became more structured in addressing these three major dimensions of capacity development (see [10]). These dimensions were each described and participants were asked to "describe your organization's current capacity to promote physical activity" given each definition. Further interview questions asked informants to discuss their organizational successes, barriers/challenges and suggestions for enhancing their organizational capacity. Discussing what might improve organizational capacity served as an indirect way for participants to think about potential dimensions of capacity in general.

### Data Analysis

Interview data were analyzed using a thematic analytic approach. Thematic analysis is a process of induction that involves the identification, coding, and organisation of themes arising from the raw data. The audiotaped interviews were transcribed and scrutinized by the first two authors through a process of close reading in order to be immersed in the data and understand participants' perceptions. Key ideas or recurrent themes were initially listed and inductive analysis was used to identify, code, and organize themes arising from the raw data with quotations serving as units of analysis [15,16]. Interview transcripts were broken down into discrete units by the second author and coded within each transcript with each based on the actual words of the respondent. These codes, tagged by initials, page and line numbers, were then examined and organized into themes with the three dimensions of capacity serving as an organizing template for categorising the emerging themes. This process was assisted by using the program QSR NVivo 8. This program supports the process of coding data in an index system with each unit of text indexed to a specific thematic, index heading. Additionally, this index system also facilitated the searching of text for patterns of coding and complemented the use of the constant comparative method [17] in comparing and contrasting each coded data unit to allow categorization into new themes. Using this constant comparison approach across responses from all of the informants assisted in the development of "plausible interpretations" from the data [18] regarding the most frequent subthemes. The subthemes concerned informants' discussion of capacity in specific relation to each of the three dimensions of will, infrastructure and leadership.

As recommended by Guba and Lincoln [19], a number of strategies were employed to enhance the trustworthiness of interpretations in terms of credibility, dependability, confirmability, and transferability. Credibility was developed through purposive sampling and regular peer debriefing among the first three authors, and then among the rest of the research team in continually reviewing and discussing findings to examine alternative possibilities, and ensure informant perspectives were appropriately reflected in the results. Dependability was primarily developed through a form of investigator triangulation in which a subset of interviews (10%) was coded by the first author to assess coding dependability [20]. Coding was found to be consistent although there were instances where discussion was required to reconcile differences regarding the centrality of certain themes that were coded. Specifically, broader themes were identified that captured informants' perspectives on their expectations of the new ParticipACTION and the challenges facing this re-launched organization. For ease of presentation, these perspectives are not reported here.

In an adapted form of member-checking, credibility and confirmability were further developed by sending a draft report of the findings to each informant inviting comment regarding how well the findings captured their own experience and any further observations that might be important to the interpretation of the findings. Although there are concerns with the notion of member checking [21], this is an important step for us in knowledge exchange and sharing the findings with participants. We received three responses commenting positively on the paper. A further criterion is transferability and the degree to which the results of qualitative research can be generalized or transferred to other contexts or settings [22]. By describing the key themes with extensive quotes from participants we hope to provide sufficient detail here to allow the reader to consider whether or not the experiences reported here are transferable to their own contexts related to physical activity promotion.

## Results

The results are presented by first summarising findings related to each of the three capacity dimensions (will/policy making; infrastructure; and leadership) and then identifying the specific subthemes related to each.

### Will/Policymaking

The current research found high levels of will and motivation to engage in physical activity promotion. Over ninety per cent of participating organizations have some mandate to promote physical activity and/or are involved with chronic disease prevention. In this section, we discuss how 'will' to promote physical activity was best characterized by participants through their explanations of policymaking successes; and by a broader societal and political climate interested in physical activity initiatives. These conditions have in turn facilitated the broadening of some key organizations' mandates to include physical activity initiatives. However, some challenges remain in policy development illustrated by gaps in school policies across the country, and at a more comprehensive level, a lack of a national physical activity platform or policy.

#### Existing policies/strategies

Overall, organizations described achieving key aspects of their mandate by having policies and strategies in place; many others understand that "with policy comes funding" (N5) and have clear plans to pursue these avenues. Several respondents described provincial-territorial policies that have assisted in meeting the 2003 Federal-Provincial/territorial government adoption of the goal of a 10 percentage point increase in the prevalence of the population in each jurisdiction (province and territory) reporting participating in regular physical activity by 2010 (see the Integrated Pan-Canadian Healthy Living Strategy at http://www.phac-aspc.gc.ca/hl-vs-strat/index.html). Flowing from these policies are plans for delivering new physical activity programs whether through non-government partners or municipalities. There was awareness and interest in setting policies to contextualize and focus physical activity efforts. This might be indicative of a wider climate where physical activity and obesity was perceived to be on the 'national agenda'.

#### Wider climate to increase physical activity

Informants believed that there was general recognition of the need for addressing physical inactivity: "huge political recognition of this is sweeping the nation" (P10) with a "groundswell" of support, in part based on "staggering health care costs." The increased physical activity focus and perception that obesity was on the national agenda was also tempered by critics of the 'obesity' crisis:

There's much more appreciation [of physical activity] - not enough, but there is more. So there's more attention at the community level. Sometimes, unfortunately, it's around the obesity issue, whereas I think they should more be focusing on the physical activity piece. (P18)

Increasing societal recognition of the importance of physical activity has in part meant that a growing number of organizations have extended their mandates and business plans to be more inclusive of participants from a wide range of physical activity backgrounds. Two competitive sport-focused organizations described their efforts to attract and support a wider base of participants. This inclusivity was partly explained through the adoption of the generic Long Term Athlete Development (LTAD) model, known as Canadian Sport for Life by federal-provincial/territorial ministers. Participants described LTAD's concept of 'Playground to Podium' in broadening the definition of physical activity and sport to encompass sporting excellence as well as overall well-being http://www.canadiansportforlife.ca.

Will and motivation for physical activity promotion were evident in four large national organizations with a limited physical activity mandate. Interview discussions emphasized physical activity promotion as a low priority in their formal mandate, but had selected it as a high priority in the survey. The discrepancy was best explained in how respondents discussed the role of physical activity within their broader organizational mission:

Only that one of our key strategies is a healthy population. So that anything we do - physical activity is not specifically mentioned in our broad mandate, because it is a very broad mandate, but it comes under our key result areas, like healthy population....we'd like to be involved though our capacity is fairly limited, but certainly the will is there, and the belief in the importance of ParticipACTION is there" (N16).

#### Policymaking challenges

Although policymaking has made positive steps at all organizational levels, supported by the increasing recognition of the importance of physical activity, respondents were concerned about government mandates that may shift with political party changes. Disjointed physical activity school policies were a recurring theme across all organizational levels. The majority of respondents highlighted concerns within the school system, complicated by variance across the provinces and territories. One national organization working closely with the educational field cited the jurisdictional challenges, as education is a provincial responsibility. Common challenges included the loss of physical education specialists and the ongoing neglect of physical literacy against core subjects. A few participants "want to move up the scale of priority" (P16) and receive the commitment of resources to deliver physical activity/education mandates. One provincial respondent suggested that affecting change has been limited by a lack of interest at the Department of Education level. Despite the repeated calls to action on the obesity and inactivity issue, participants stressed that school and community access policies have been an ongoing battle for many Canadian jurisdictions and requires more collaboration and strategic thinking between school boards and municipalities. Repeated calls were made to advance the physical activity agenda through some type of national strategic partnership.

#### Need for national leadership

One-third of participants explicitly requested some form of national guidance, leadership, and/or platform for the physical activity field. They envisioned a national physical activity policy document that integrates some of the best and innovative ideas currently being developed in the field. One respondent captured this common theme:

There's no national policy for physical activity, which would be enormously helpful. We really are working in isolation...there's a lot of form and consistency around sport action across the province because of the sport policy [Canadian Sport Policy; http://www.pch.gc.ca/pgm/sc/pol/pcs-csp/index-eng.cfm], it's fantastic. The physical activity side: people are all over the map. (P2)

Two national level organizations with a limited physical activity mandate offered an "outsider" perspective: "The other greatest barrier is their lack of public policy, supporting that, and some political leadership" (N4). Another made reference to a "disconnect" within the physical activity field, especially in advancing the Coalition for Active Living (CAL) Physical Activity Strategy (2005):

...it's a document that isn't really geared toward anybody in particular, it just says, "this is what needs to be done", and they're not good at saying to the federal government "here's what you should be doing." (N17)

Overall, organizational will is strong among all organizations with some concerns for the intricate jurisdictional issues in the educational sector: participants pointed to the need for more collaborative partnerships and strong leadership at the national level.

### Organizational Infrastructure

Infrastructure, the second capacity building element, is of critical concern to organizations as they deal with the complexities and idiosyncrasies of funding, human resources and knowledge transfer. In this section we discuss how infrastructure was primarily considered in the context of financial and human resources. This required expertise in accessing funds in the context of funding instability, and concerns about shifting funding priorities. Funding challenges could be partly addressed given existing human resource strengths although difficulties in staff retention remain.

#### Financial and human resources

Participants tended to focus on current financial and human resource capabilities, a common finding in the health promotion literature [12,23]. For example, one participant described:

It's really about the financial and the human resources and the two of them are interlinked: we can't hire more staff without more financial resources. That's the bottom line. And even if we had a whole bunch of financial resource given to us, if it had some kind of restriction on it in terms of not being able to hire people, it wouldn't do us any good because we're maxed out right now from a human resource capacity perspective. (N10)

Overall, current organizational infrastructure capacity exists on a broad continuum with some organizations describing a safe or sufficient level of funding and others facing the threat of receiving decreased amounts or losing all funding. In effect, most organizations described adequate financial and human resources because organizations have learned to be creative and effective with available funds and staff (both paid and volunteer). This efficiency is illustrated here:

If I would describe the sport world, we are one of the most efficient groups of people who use money. In fact, our efficiencies in using money have been to our detriment, because we've learned to live with what we have, no one ever thinks to say that we need more. (P4)

Funds have been generated from a range of sources but overall many respondents discussed the funder instability that is making it increasingly difficult for organizations to effectively deliver their physical activity mandate. Funding challenges are further hampered by increasing competition for money between like-minded organizations.

#### Accessing funds

Organizations have been competent in accessing needed financial resources from a wide range of government and agency sources, including, "outside of what might've been the normal points of engagement" (N1). Government partnerships comprise a range of funding support including federal departments such as *Transport Canada *and *Industry Canada and, Citizenship & Immigration Canada*, alongside bilateral funding agreements between provincial and federal governments. These and other governmental supports have provided secure funding and enabled organizations to carry out support for provincial and local groups and organizations. Other fund generation has come from organization memberships, workshop delivery, and online sales of resources:

...we give them out [resources] and encourage people to pick it up. At some point, though, we need to get money back from it...people have to pay for it, but everybody likes them for nothing. (N11)

Participants did express reservations about relying too heavily on one government funding source: "the idea in diversifying the funding base is that it helps to alleviate that pressure [short funding cycles] a little bit; but it's definitely a big pressure" (N8). In addition, another participant argued that:

It's at the whim of the government as to whether the funding comes in...the government changed hands and of course they can't agree to do exactly what the last government did. They have to review everything, which is typical, so there was no funding for - I don't know if it was 6 or 8 months or close to a year. (N6)

Corporate sponsorship is an increasingly important aspect of several organizations' funding sources. One successful national level respondent explained this approach to generating funds:

Well, we've been successful in getting corporate sponsors on board and we do that on a program basis. We don't just go out and ask them for money...we research them and we find out what their corporate objectives are in either their marketing or sponsorship department or their community investment department...we've got to invest some time and effort in people to go find it. (N5)

Sole funding from the corporate sector was also identified as a concern in that corporate mandates may not fit the organization's goals/mission, or these funders may only support programs that can show their brand or logo, with little or no interest in advocacy work.

#### Instability of funding

Despite securing these funding sources, the "instability of funding" (P7) prevailed as a key concern for the majority of participants. Funding priorities and competition between organizations created some tension within the physical activity promotion environment further hampering research and marketing activities and initiatives. Funding cycles created some concerns including the retention of skilled staff in the face of funding constraints:

Funding is always a challenge, especially with the timing of the funding...and when we're looking at one-year funding for a program, it makes things really difficult...And, come April 1^st^, we'll be back in the same situation again. So it's kind of like a vicious cycle, a bit. ...So you kind of want to keep yourself in a situation where you can survive the blips a little bit, because I guess my biggest fear was that I never wanted to have to let a great staff person go because of funding constraints. (N8)

Financial security was threatened with money often allocated for "program, project or grant specific" initiatives (N15) and "specific deliverables" (P1). Although contribution agreements are a welcome funding source, all money must be spent on project deliverables: this "absence of core funding" (N18) has meant that no funds can be spent on operating costs. The extensive proposal writing and reporting for each amount of funding received, whether small or large was highlighted: "We spend a lot of our time putting in a proposal, and then it can takes months and months to get a response back" (N8):

Then especially with some of the smaller funding pots, they require that you send those quarterly reports, which are just as long as a larger funding pot to do the quarterly reports. So, when the community's looking for program delivery and you're sitting in your office reporting on $1000, you start to question what you're doing. (L5)

Funding insecurity also makes it difficult to provide sustained interventions:

It's easy enough to get something that's a six month or one year kind of grant or project funding, but if we're wanting to make changes in behaviour and change attitude, it's very difficult for non-profits these days to access long-term funding, or funding that can allow us to commit a longer period of time to an intervention. (P8)

#### Funder priorities

When discussing a range of funding issues, informants identified a discernable shift in funder priorities. There was the perception that emerging child/youth physical activity priority areas were squeezing funding for a few organizations with different mandates. Additionally, strong competition amongst sport and recreation groups to procure funds from outside funders may be viewed "as having too many hands" in the field (P7). In general, a lack of funds was routinely cited by many organizations for not conducting formal evaluations of programs or organizational processes. Furthermore, funding instability also influenced physical activity promotion and social marketing costs:

We don't have the resources to do it; I think we got an estimate one year, it would cost us close to $400 000 for the first year, $200 000 for the next three years to do a social marketing campaign. And just the dollars involved with that; then it's only provincial. (P17)

An organization in another province was facing similar issues:

"The research, the creative work, all of that stuff is astronomical in price, and we're all trying to do it on our own. If I could keep that half a million dollars that we spend on social marketing development and put it into programming, we would be so ahead" (P14).

#### Human resources

Across all organizations, human resource strengths included enthusiastic and skilled staff, which enabled effective delivery of mandates and organizational objectives. Human resource challenges included staff retention and training, and time management demands. The majority of informants praised their committed volunteer and paid staff and viewed these relationships as key to their successful operations. Various responses described this commitment, expertise and experience: "hard-working volunteer executive" (P13); "local/internal champion" (P16) and; "passionate" (P20). Many organizations relied on volunteer staff:

We have tremendous people across the country with a variety of different backgrounds and knowledge, and they're all prepared to volunteer that knowledge and their time for the development of whatever the project is. So to me, that's a huge strength, because we have a very well-respected board and a research committee. (N6)

A particularly important aspect of human resource capacity was supporting "local champions." By achieving "community buy-in" (L6), local groups were able to deliver and sustain physical activity promotion initiatives. One provincial funder argued that skilled persons are central to local human resource capacity and require strong support:

So what we've done is, anytime that we want to impact or influence behaviour in communities...one of the things we've heard time after time is that we have to sort of work with those community champions...give them all of the possible supports we can, so that when they effect change at the community level, a) they're informed, b) they have all the tools they need, and c) there is somebody standing behind them from a regional or territorial level. (P10)

Providing heavy subsidies, for example, to coaching and leadership training ensured that one local community gained the necessary expertise and continued the local commitment to physical activity initiatives. Such efforts are ensuring that community-based, long-term physical activity strategies are rooted in local needs: "it's giving them some arms and legs to be able to do things" (P18) and "it still means the folks locally have to take it on and embrace it" (N11). Similarly, research on heart health promotion initiatives in three Canadian provinces found that increased staff focus on community mobilization and ownership to sustain activities ultimately served to enhance local organizational capacity [14].

#### Staff retention

In some cases, funding challenges resulted in staff layoffs, difficulties in retaining skilled persons and few funds for volunteer training. Remote and/or smaller communities have difficulties finding and maintaining skilled staff to work in physical activity promotion, including the challenge of volunteer training:

We're having some success when it's a local person from the community that takes on the role, but often they don't have the education background...but it's a high-profile position...and you really need the training behind you to have that base, so often that's a problem, too. (L6)

Time management and "multiple demands" (N4) were common concerns for both paid and volunteer staff. Participants discussed time constraints differently with one organization having, "little time to think" (P13) and cited repeated delays in their plans to review their mission and goals; multiple duties and a concern with delivering "practically-oriented" activities prevailed. One small organization found it difficult to keep an "eye on the entire physical activity environment" (N13) and figure out what will be the next physical activity trend.

Although volunteers are key to organizational success, several important concerns were expressed: "We're dealing with community sport people here, they're overworked, they volunteer (it's the 80/20 role, they do 80 per cent of the work and it's only 20 per cent of them doing it), but they believe in it" (N14). In addition, highly-skilled people in small communities are described as "very busy," juggling multiple roles, and needing support:

And how can you go mobilize and advocate the activity if you don't have people to do it? And it's not fair to place that burden on volunteers....that's a capacity that needs to be improved, not just nationally, equally as well provincially. (N11)

### Leadership

Leadership is the third aspect of organizational capacity. Strong and effective leadership was considered a means to counteract financial and human resource shortfalls. Leadership issues were often highlighted in discussions of organizational successes and barriers:

We learned very quickly that we simply didn't have the capacity... a strong understanding of the issue and the breadth of the issue, a strong understanding of what really needed to be done to address the issue. So really, leadership capacity. We had resources, we were given really good financial resources, but really we didn't have the leadership. (P2)

This comment introduces both the importance of leadership capacity and its complexity, in part because of links between human and financial resources. Overall, the majority of national organizations viewed their current leadership ability in positive ways with strengths in a wide variety of areas. In this section, we will discuss how leadership was described in terms of productive and collaborative partnerships although such partnerships were not always without strain; and through developing a greater role in terms of advocacy.

#### Partnerships/collaborations/networks

Without strong leadership, partnerships can be difficult to develop. Robinson and colleagues [14] discuss leadership capacity through partnership number, and organization type or nature. Joffres and colleague's [12] cooperative and collaborative partnerships also hold some resonance here. Our findings suggest an emerging trend towards collective work amongst a wide range of like-minded sport and physical activity organizations in Canada. This means that more "cooperative strategies" have been employed to achieve goals in this area (e.g., policy initiatives) (N1). One respondent noted: "The only way we function as an organization is through partnerships...that's always how we've gotten stuff done." (N10)

There were existing inter-organizational partnerships that drew across a number of sectors and mandates. Partnerships with organizations sharing physical activity mandates and targeting a wide range of issues such as disability, aging, youth, girls and women, physical education, and tourism were common. Broad federal government departments and agencies have been increasingly joining physical activity initiatives (e.g., active transport). Partnering with organizations having non-specific physical activity mandates, "like-minded health-related organizations" (N8) have occurred and were described in this way: "We're a member of the Chronic Disease Prevention Alliance of Canada (CDPAC) because we think we have a lot in common with those specific health charities around the whole role of promoting prevention and healthy lifestyles" (N21). One national organization commented on increasing their partnerships: "individual provinces and municipalities are doing great stuff around physical activity. We want to be better able to share what they're doing" (N22). Another large national physical activity organization partners by offering in-kind services to low-resourced organizations.

As with national organizations, provincial and local organizations also stressed the importance of having strong and extensive partnerships, a key indicator of leadership capacity. Finding people with the needed expertise, filling skilled positions, and retaining those people were difficult for rural, remote, and/or northern communities, however these effects were minimized when organizations readily shared resources. Thus, although "scrounging and borrowing" (P15) occurs in many of these communities, ultimately it is the intensive partnering efforts that prevail and aid in achieving physical activity mandates.

Several participants described the "positive" developments, working as "co-partners" on various physical activity initiatives inter-sectorally and inter-departmentally. One physical activity initiative engaged a "team approach" with education, health, recreation, sport, downtown business, and urban planning: "so they all come together and develop and implement these strategies and we support that" (P2). Two participants reported on strategic partnerships between previously considered disparate groups that never would have occurred five to ten years ago. In more remote and/or rural locations, evidence of a more cooperative approach to physical activity promotion and delivery, and existence of overlap between local and provincial designations occurred:

I see them often, I know who they are, I can access them almost at any time, and they can do the same with me. but I think it's much more complicated, because even though you need each other, you're small, and both human and fiscal resources are limited, you still need to provide the same amount of service as the other big provinces do, so you're a little top-heavy on human resources...so, every time you want to go forward with a particular project or initiative, you absolutely need partners. (P14)

All of these partnerships suggested strong potential leadership capacity in effectively delivering physical activity initiatives and setting the stage for further policy development and implementation.

#### Partnership challenges

Strategic partnerships and collaborative activities have been hindered by a lack of coordination at local and provincial levels and from a larger perspective, a dearth of national leadership. Some participants stressed the importance of working more closely in efficient program delivery and ensuring that the physical activity/inactivity message is not diluted. Disorganization in the broad physical activity sector was noted through several comments: " [a] need for a better coordinated approach" (N21) and "it's still a bit of a struggle to find common ground that everybody can buy into. But we're starting" (P18). To address these coordination problems people need to "connect with, on a regular basis, the multitude of organizations that are working at a national level on the issue, we're all doing a lot of things and there isn't really a forum where we come together to discuss what we're doing" (N17):

Sometimes we're [working] in silos. Sometimes, the sport community and the recreation community and others can come together and agree that this is what we ask; we may all want to see physical activity levels increase or physical inactivity levels decrease, but what we're asking for is different...if we were together, we would be greater than the sum of our parts...but unfortunately, it just becomes that we're kind of stuck on our agendas. (P8)

Similarly, repetitious programs could be streamlined through strong national leadership: there's "too much overlap in physical activity organizations across Canada":

Individual provinces and municipalities are doing great stuff around physical activity. We want to be able to better share what they're doing... [but] every one of the provinces...we're all doing the same thing...why wouldn't we, instead, work together and say "you, [province], do this, you [province] do this," and then we'll all sit back together and look at the results and then share the success. Instead, we can spread out the very small amount of money that physical activity ever has and use it more efficiently. (N22)

#### Advocacy roles

Strong organizational leadership qualities were also exemplified by organizations taking on advocacy roles, whether engaging in activities to change rules of governments or organizations, or other responsible bodies [12]. Key stakeholder partnerships have emerged in advocacy efforts: "we've also begun to really work well with some of our provincial governments, around playing a role around coordination of public policy agenda" (N21). Several respondents expressed caution in partnering with non-physical activity organizations: a central focus on activity could be diminished or lost.

We're more on now to chronic disease, healthy lifestyles, and everything else, and we've lost the mass physical activity and active living voice out there...so now people look at us "well they're part of chronic disease, they're part of healthy lifestyles, which includes nutrition, smoking..." and there's no one out there speaking on behalf of physical activity. (P17)

The physical activity field is increasingly viewed through primarily a health care lens and participants feared that they "won't get the ear of government" (N19). Advocacy leadership was suggested to counter what a few participants felt were competing health mandates (e.g., nutrition, tobacco, workplace health and safety) that may ultimately drive physical activity to the sidelines. That is, advocacy was required to ensure that there was a loud and consistent message being communicated to Canadians about physical activity that was not lost within broader health communications. In addition, the increasing focus on the obesity epidemic has had unexpected results when physical activity is meshed with nutrition and eating disorders through partnerships where, "everything falls back to obesity" and there exists a tendency for people to get drawn into the obesity management area. Strong advocacy is required for physical activity, rather than obesity-driven agendas:

We [physical activity] can shoot ourselves in the foot for two reasons: we can come in direct conflict with those in the disordered eating community because of the different messaging...to me if you come with the message that physical activity is good for kids of all weights, shape and size, that's ok, that's it, full stop. We don't need to get into these debates about how many fat kids we have or don't have - physical activity is important, period. Yes. Joy is important. The approach that we're taking in the field is based on fear and it makes me very nervous because if we go back to taking a very prescriptive role we'll just come full circle again. (N2)

The increased focus on obesity ultimately may threaten the goals of people working in active living and recreation:

...how the government uses physical activity as a means to an end rather than an end in itself. If all physical activity exists for is to reduce health care costs and to reduce obesity, then the barrier in itself is that it's only seen as a means to an end and not valued for the experience. (P8)

The challenge identified by participants was that advocacy is rarely funded in organizations, not viewed as a deliverable, yet has been deemed by most organizations as a necessary component to effectively promote physical activity:

We don't have programs. What we're trying to do is the basic grunt work of the physical activity field, and that's not sexy to people. They don't want to fund infrastructure, they don't want to fund staff. They don't want to fund the basic underpinnings of how things are coordinated. They don't get any bang for their buck. (N22)

As noted earlier, several organizations mentioned that a national voice for physical activity could play a central role in advocacy efforts targeted at all levels of government and in myriad settings (schools, workplace, home, communities). The new ParticipACTION could play that role.

## Discussion

The purpose of this study was to qualitatively explore baseline organizational capacity to promote physical activity messages, programs, and services within the Canadian context. It is one of the first efforts to adapt health promotion capacity models to the field of physical activity and explore baseline organizational capacity to promote physical activity concurrent to the launch of a large-scale, national physical activity initiative. Overall, the organizational capacity model described by Smith and colleagues [10] was appropriate in framing current organizational strengths and challenges within the Canadian physical activity field and may be of use to international researchers investigating capacity elsewhere. Although the concept of "organizational capacity" was often perceived as vague by many informants, prompts in relation to will, infrastructure, and leadership added clarity to these discussions. Respondents enthusiastically shared organizational successes, challenges, and capacity strengths.

### Capacity Strengths

In line with the results reported by Plotnikoff and colleagues [7], current capacity to promote physical activity in Canada could be described as good. Informants described a number of organizational strengths. A concentrated commitment to the physical activity field was evident in the wide range of policymaking efforts occurring across Canada, in part supported by the wider societal climate focusing on healthy initiatives and recently developed federal strategies and policies in both healthy living and sport. Such policy frameworks have enabled increased funds and other resources to flow into important physical activity initiatives. At the federal level, Barclay [24] found that the Canadian Sport Policy http://www.pch.gc.ca/pgm/sc/pol/pcs-csp/index-eng.cfm and Healthy Living Strategy http://www.phac-aspc.gc.ca/hl-vs-strat/index.html were deemed "hopeful signs" within Sport Canada and Health Canada (page 7) and our study suggests these federal directives are enabling organizations to address their mandates.

Experienced leadership was a central factor in effectively delivering physical activity mandates. As a result of this leadership, there exists across Canada local, provincial and territorial partnerships working alongside key network and coalition groups. This has meant that complex physical activity issues are now being addressed, albeit not without some strain in these partnerships. The findings on partnership efforts also corroborate aspects of Interorganizational Relations (IOR) theory [25] in that socio-economic and political factors have been intricately woven in physical activity and health issues. These issues require partnerships between a wide range of sectors and at all levels, ultimately working to achieve policy objectives [26]. For example, informants in our study highlighted innovative initiatives that illustrated such intersectoral partnerships (e.g., Commuter Challenge, active transport/built environment/smart-growth community initiatives). Resource sharing and resource acquisition [14] were also evident and web-based sharing and receiving in-kind services from more resourced organizations were described. Research partnerships have further enabled organizations to attain relevant and recent data to inform policies and practices, albeit not always relevant to local conditions and situations.

### Capacity challenges

Although human resource successes have been due largely to committed people working in both volunteer and paid roles, the physical activity field has been fraught with financial resource difficulties. Funding restrictions have negatively affected staff retention and training opportunities, further exacerbated by the multiple demands on staff which are often more pronounced for small, remote and/or northern communities. Several respondents focused on the difficulties of unstable funding.

These concerns are reflected in other studies on the non-profit sector and funding practices [27,28]. Eakin's [27] study was similar to our findings regarding the unreliability of funding as she explains: "funders are slow to approve/reject grants, and the slow response time causes 'gap' problems for service delivery" (page 4). Eakin concludes that "The administrative burden by funders on community non-profit organizations is so heavy and so unrelenting, and places so many constraints on their ability to operate that it is a wonder they can deliver any services effectively" (page 3).

The current research also uncovered problems with funding reallocation from a population focus to current child and youth physical activity and obesity initiatives. An increasing number of organizations reported a limited capacity to meet their goals in the wake of such funder decisions. In contrast to the findings reported by Plotnikoff and colleagues [7] where educational organizations appeared to have greater capacity than other organizations, the majority of key informants (notably, most were not in the educational sector) expressed concerns with the inconsistency in school physical activity policies across the country. It was suggested that the potential role of schools could in part be bolstered by a more comprehensive national physical activity platform/strategy or policy. Such a national, multi-sectoral policy could result in a more coordinated approach to physical activity and provide pressure for a physical activity-centred agenda (see [29]).

Participants suggested that organizations should proceed cautiously when selecting health partnerships because of the fear of losing key physical in/activity messages. Some did not wholeheartedly support obesity-centred research and program initiatives in the physical activity field. Participants in Barclay's 2003 Public Policy Forum paper [24], *Finding Common Ground*, corroborate some of these concerns in alleging that Health Canada places its lowest priority on physical activity promotion within the already secondary context of health promotion. Our findings highlight the fact that participants have similar concerns, with some participants critically examining the increasingly prescriptive approach to physical activity. Although collaborative work with other health mandates was welcomed, the prevailing concern was that physical activity is generally at a low priority in the larger health context and continued advocacy was required.

## Conclusion

Key informants in this study painted a generally positive picture of the current organizational capacity to promote physical activity in Canada. Will and leadership were clear strengths while financial and human resources remained the greatest concern. However, it may be that only those informants with generally positive perceptions agreed to participate in the study. Future evaluations could consider a range of indicators for monitoring how existing organizational capacity was utilized or indirectly enhanced by ParticipACTION, or other physical activity initiatives in the coming years.

A first indicator could be the creation of a national physical activity policy or framework in the coming years. Such a public policy is expected to elevate the status of physical activity within the broader public health and health care climate and may positively impact the development of more comprehensive provincial physical activity policies for school settings. Included in this exploration would be tracking the process by which such a policy was developed: who were the major stakeholders (groups/organizations and/or individuals)?; what was the major impetus for the policy creation (political and health conditions and/or forces at the time)?; what other policies may have been influential during the policy-making process?; has the policy been implemented in the intended manner?, and; what efforts were in place to monitor the policy's effectiveness? Assessing these factors would allow researchers to determine if various aspects of capacity led to development of policy in a concerted way or whether policy was developed independent of capacity.

A second indicator might be the examination of partnerships, deemed by participants in our study as a necessity in physical activity capacity building. In line with IOR theory [25], monitoring partnership shifts over time is critical for fully exploring the process of capacity building. Examining existing partnerships through qualitative and quantitative means may uncover the following: what networks or coalitions remain, changed or have become obsolete during this period?; what role will private/for profit organizations play as ParticipACTION embarks on securing broader bases of funding support (only one for profit participant was recruited for this study, a limitation that should be addressed in future research), and; what role did existing or new partnerships play in the creation of a national policy or platform for physical activity?

A third indicator of growing national organizational capacity could be the changing contributions of large health organizations without explicitly defined physical activity mandates to the field of physical activity promotion. For example, based on their self-described strong organizational capacity, have they been invited to play a role in promoting physical activity? Specifically, how or will such organizations be engaged to commit long-term to the physical activity field? In what way and by whom?

Since funding issues were a central concern for participants in this study, a final indicator could track funding process reform, as Eakin ([27], page 44) explains, in terms of "simplifying application processes, reducing the frequency and complexity of reporting, and speeding up the turn around time for grants". In the long-term, funding reform could minimize the administrative burden many organizations experienced. A sub-question to assess is the fate of organizations that experienced either reduced funding or ceased operations entirely. Have they been reinstated? If not, what gaps remain or have been addressed in physical activity promotion (e.g., subpopulations such as older adults) during the next decade? Furthermore, human resource issues should be assessed in light of funding constraints directly impacting on this area, most notably in the monitoring of paid and volunteer staff retention and the steps taken to alleviate these concerns in more remote or rural communities.

These indicators could form the basis for future evaluations of organizational capacity for promoting physical activity in Canada. Ultimately, a longitudinal evaluation will uncover whether ParticipACTION or other initiatives successfully enhance or utilize existing organizational capacity to mobilize and advocate for a more active Canada.

## Competing interests

None of the authors have any competing interests. LRB and LG are members of the Content and Collaboration Committee of ParticipACTION. MT was a founding member of the Board of Directors of the new ParticipACTION.

## Authors' contributions

GF, CM and RCP contributed to the design, data collection, data interpretation, and the drafting and the coordination of the manuscript. All other authors advised on the design, provided input at each stage of the manuscript draft and read the paper critically for theoretical content and interpretation of findings. All authors read and approved the final manuscript.
